# Case report and literature review: primary hepatic leiomyosarcoma with misdiagnosis and SDHB positive expression

**DOI:** 10.3389/fonc.2025.1675617

**Published:** 2025-11-17

**Authors:** Chengbin Lu, Wenchao Ding, Xiaoli Ma, Benmo Xu, Zongyan Luo, Can Li, Sinan Yang, Jinye Yang, Yuanlong Shi, Yufan Bai

**Affiliations:** 1 Department of Hepatobiliary-Pancreatic-Thyroid-Vascular Surgery, Yuxi People’s Hospital, The Sixth Affiliated Hospital of Kunming Medical University, Yuxi, Yunnan, China; 2 The Third Affiliated Hospital of Kunming Medical University, Yunnan Cancer Hospital, Peking University Cancer Hospital Yunnan, Kunming, Yunnan, China

**Keywords:** hepatic leiomyosarcoma, misdiagnosis, radiological differentiation, SDHB, case report

## Abstract

**Background:**

Primary hepatic leiomyosarcoma (PHL) is an extremely rare malignancy often misdiagnosed due to nonspecific imaging features overlapping with cholangiocarcinoma. This case highlights diagnostic challenges and the potential association of SDHB positivity with PHL prognosis.

**Case Presentation:**

A middle-aged male presented with an asymptomatic liver mass. Preoperative CT/MRI revealed a 10-cm lesion with progressive heterogeneous enhancement and delayed capsular enhancement, initially diagnosed as cholangiocarcinoma. Left hepatic lobectomy confirmed PHL. Immunohistochemistry showed SDHB positivity and low Ki67 (15%). The patient recovered well with no recurrence at 6-month follow-up.

**Conclusions:**

PHL can radiologically mimic cholangiocarcinoma, necessitating inclusion in differential diagnoses for “cholangiocarcinoma-like” liver masses. SDHB positivity may indicate favorable tumor biology, but further studies are needed to validate its prognostic value. Surgical resection remains curative for localized PHL.

## Introduction

Leiomyosarcoma (LMS) is a rare malignant tumor primarily originating from smooth muscle cells, accounting for approximately 11% of all soft tissue tumors (1, 2). This tumor can occur in the uterus, gastrointestinal tract, or blood vessels, but hepatic involvement is uncommon. Primary hepatic leiomyosarcoma (PHL)—an even rarer malignant liver tumor—often presents with atypical clinical manifestations, leading to imaging misdiagnosis. Typically, PHL originates from smooth muscle cells in the liver. Despite its low incidence, patients with PHL often have a poor prognosis once diagnosed (3). Although advancements in imaging technology have improved diagnostic accuracy, misdiagnosis remains a significant challenge, particularly in the case of PHL. The imaging features of PHL frequently resemble those of benign liver tumors, contributing to diagnostic errors (4, 5). Therefore, accurate imaging assessment and careful differential diagnosis are essential for improving the early diagnosis of PHL. In this case, positive expression of SDHB (Succinate Dehydrogenase B) was detected. Previous studies have shown that the expression of SDHB is closely associated with the tumor’s invasive behavior and metastatic potential, particularly in PHL. The absence or mutation of SDHB may be linked to the malignancy and prognosis of the tumor (6). The positive SDHB result in this case may offer specific insights into the tumor’s biological behavior and prognosis, warranting further investigation.

This case report presents the diagnosis and treatment of a rare case of primary hepatic leiomyosarcoma (PHL). The patient initially presented with a liver mass, which was misdiagnosed as cholangiocarcinoma based on preoperative imaging. However, after undergoing a left lateral lobectomy, the diagnosis was confirmed as PHL. The distinctiveness of this case lies in the large size of the tumor (10 cm × 8 cm × 7 cm) and the patient’s favorable postoperative recovery. The significance of this case lies in providing clinicians with a new perspective for differential diagnosis, particularly when encountering liver masses, where PHL should be considered as a potential diagnosis.

## Case presentation

### General information

A middle-aged male patient was admitted to the hospital following the discovery of a liver mass four days earlier. He did not exhibit typical symptoms such as abdominal pain or jaundice. His medical history included previous hernia repair surgery and excision of a subcutaneous lipoma; he had no prior history of liver disease, family history of malignancies, or exposure to carcinogens. Physical examination revealed a flat abdomen with no tenderness or rebound tenderness, no palpable masses, a negative Murphy’s sign, and normal bowel sounds. An abdominal ultrasound conducted at an external facility revealed a hypoechoic mass in the left lobe of the liver, along with slightly hypoechoic nodules within the liver. An MRI performed at another hospital identified a large space-occupying lesion in the gallbladder fossa with ill-defined boundaries between the lesion and the left lobe of the liver. Additionally, multiple small cysts were noted in the right lobe of the liver, and a small cyst was observed in the tail of the pancreas.

### Timeline of clinical events

The key milestones of the patient’s care are summarized in **Table 1**.

**Table 1:** Summarizes the key milestones of the patient’s care.

Date	Event
Dec 12, 2024	Routine physical examination detects liver mass; no symptoms
Dec 16, 2024	Completes upper abdomen CT, MRI, ultrasound, and tumor marker testing
Dec 20, 2024	Preoperative evaluation confirms eligibility for left hepatic lateral segmentectomy
Dec 23, 2024	Undergoes surgery; intraoperative frozen section: hepatic spindle cell tumor
Dec 28, 2024	Postoperative pathology confirms PHL; discharged (liver function normal)
Jan 23, 2025	1-month follow-up: CT shows no recurrence; tumor markers normal
Feb 24, 2025	2-month follow-up: No abdominal discomfort; daily activities resumed
Mar 25, 2025	3-month follow-up: No metastasis; returns to part-time work
Jun 23, 2025	6-month follow-up: Contrast-enhanced CT confirms no recurrence/metastasis; SDHB expression reconfirmed

### Examination

#### CT scan

**Figure 1:**
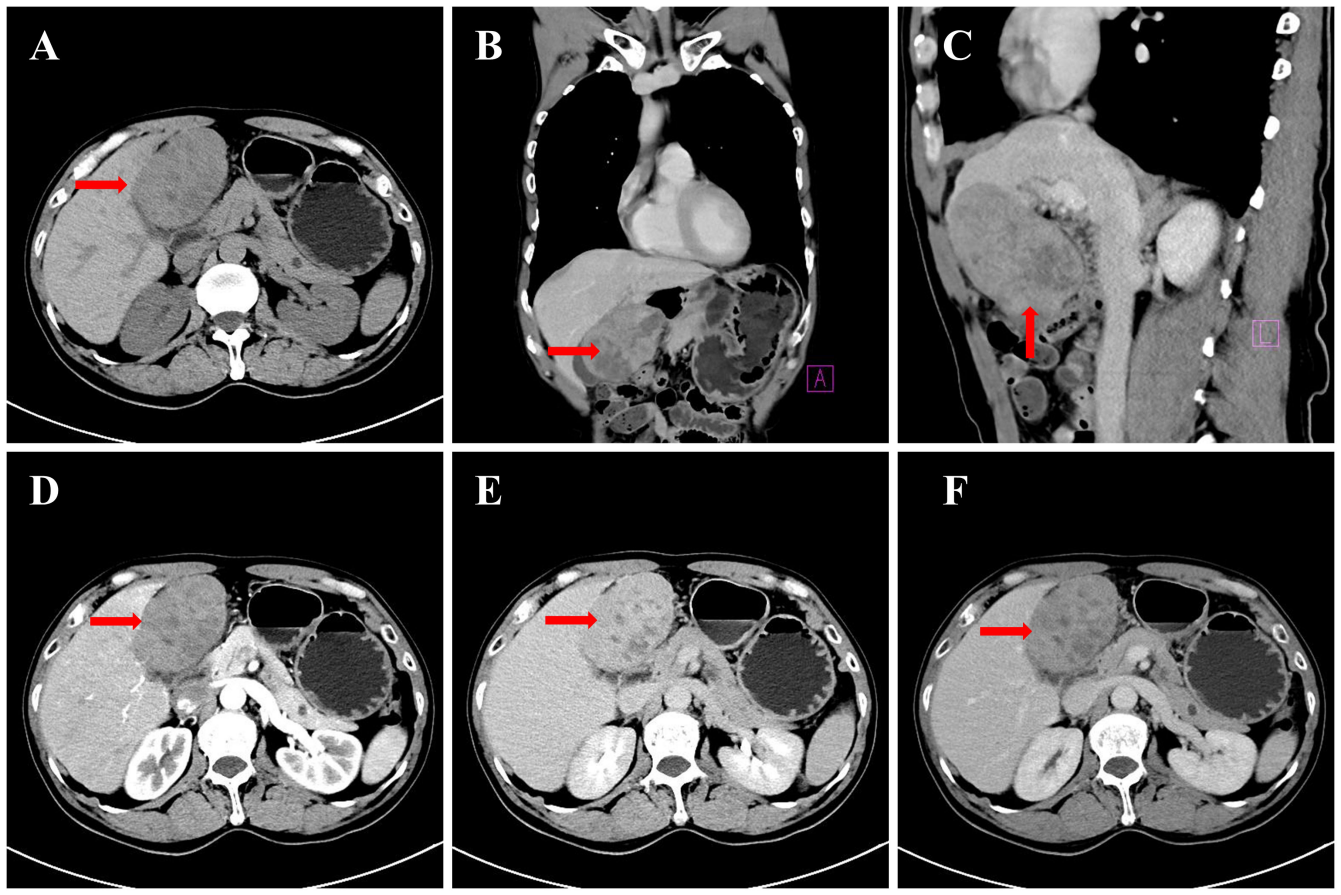
CT images of the upper abdomen: **(A)** Non-contrast axial view (left hepatic mass, arrow); **(B)** Non-contrast coronal reconstruction (mass compressing gallbladder, arrow); **(C)** Non-contrast sagittal reconstruction (mass-gastric antrum adhesion, arrow); **(D)** Contrast arterial phase (heterogeneous enhancement, arrow); **(E)** Contrast portal phase (parenchymal enhancement match, arrow); **(F)** Contrast delayed phase (capsular enhancement, arrow).

A CT scan of the upper abdomen was performed on December 16, 2024, with findings shown in **Figure 1**. On December 16, 2024, a CT scan of the upper abdomen revealed a space-occupying lesion in the left lobe of the liver. The lesion extended downward, compressing the gallbladder, with partial loss of visualization of the gallbladder wall. The lesion measured approximately 9.3 × 6.0 × 8.3 cm and was closely adherent to the gastric wall at the gastric antrum. On contrast-enhanced imaging, the lesion exhibited progressive, heterogeneous enhancement with multiple small areas of non-enhancement. In the arterial phase, the surrounding hepatic parenchyma showed patchy areas of marked enhancement, while enhancement in later phases was similar to that of the left hepatic parenchyma. No dilation of the intra- or extrahepatic bile ducts was observed. Multiple abnormal enhancing nodules were also noted within the liver parenchyma, with prominent enhancement in the arterial phase, while enhancement in the portal venous and delayed phases was unclear. The largest nodule, located in segment S8 of the liver, measured approximately 1.1 × 0.9 cm. Additionally, multiple round hypointense lesions were seen in the hepatic parenchyma, with the largest measuring approximately 0.9 cm. The pancreas appeared normal in size and shape, with a small, round hypodense lesion in the tail of the pancreas, measuring approximately 0.8 × 0.9 cm. No enhancement was noted on contrast-enhanced imaging, and no dilation of the main pancreatic duct was observed. No abnormalities were detected in the splenic parenchyma, and no abnormal enhancement was noted. No enlarged lymph nodes were observed in the hepatic hilum or retroperitoneum. The imaging findings suggest: a mass in the left lobe of the liver, consistent with a malignant tumor of hepatic origin (possibly cholangiocarcinoma), with abnormal perfusion adjacent to the liver; multiple areas of abnormal perfusion or small vascular tumors in the liver; a small cyst in the pancreatic tail; and multiple small cysts in the liver.

#### MRI examination

**Figure 2:**
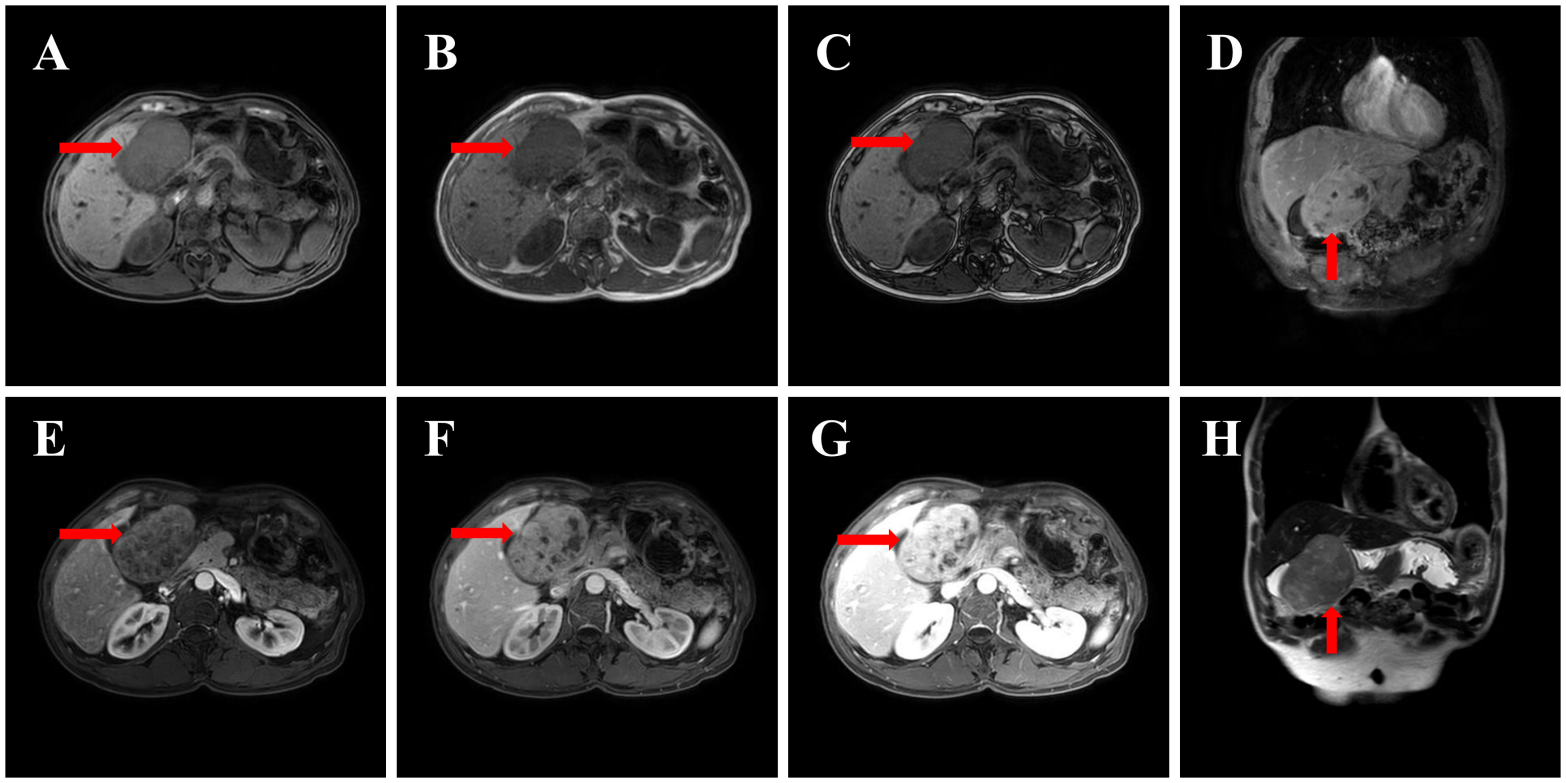
MRI image: **(A)** T1WI axial (mass isointense to liver, arrow); **(B)** T2WI axial (mixed signal, arrow); **(C)** DWI (restricted diffusion, arrow); **(D)** T2WI coronal (mass displacing gallbladder, arrow); **(E)** Contrast arterial phase (heterogeneous enhancement, arrow); **(F)** Contrast portal phase (enhancement progression, arrow); **(G)** Contrast delayed phase (capsular enhancement, arrow); **(H)** T1WI sagittal (mass-gallbladder relationship, arrow).

An abdominal MRI (including contrast-enhanced scanning and MRCP) was conducted on December 16, 2024; results are presented in **Figure 2**. On December 16, 2024, an MRI of the upper abdomen, including contrast-enhanced scanning and MR cholangiopancreatography (MRCP), was performed, revealing a round-shaped mass lesion located in the right upper abdominal gallbladder fossa, approximately 9.2 cm × 6.0 cm × 8.7 cm in size. The lesion demonstrated heterogeneous signal intensity. On T1-weighted images (T1WI), the lesion appeared isointense to slightly hypointense, while on T2-weighted images (T2WI), it exhibited slightly hypointense and slightly hyperintense mixed signals. Diffusion-weighted imaging (DWI) showed restricted diffusion. The lesion had indistinct borders with the left hepatic lobe, and it displaced the adjacent gallbladder and gastric antrum. Contrast-enhanced imaging revealed progressive, markedly heterogeneous enhancement, with the capsule showing marked enhancement in the delayed phase. Patchy enhancement of the left lateral segment of the liver was observed in the arterial phase, which was consistent with the surrounding liver parenchyma in the portal and delayed phases. Multiple round-shaped abnormal signal shadows were noted within the hepatic parenchyma, showing low signal intensity on T1WI, slightly higher signal intensity on T2WI, and high signal intensity on MRCP. No enhancement was seen on contrast-enhanced imaging. Small, round, and patchy abnormal signals were identified in hepatic segments S2 and S8, with low signal intensity on T1WI, high signal intensity on T2WI, and high signal intensity on DWI. The larger lesions measured approximately 1.0 x 1.2 cm, had clear borders, and demonstrated progressive, marked, and uniform enhancement on contrast-enhanced scanning. Normal vascular distribution was observed within the liver. No widening of the hepatic hilum or hepatic fissure, nor dilation of the intra- or extrahepatic bile ducts was present. The common bile duct measured approximately 0.4 cm in maximum diameter. A small nodular shadow was noted in the pancreatic tail, measuring approximately 0.8 x 0.9 cm, with low signal intensity on T1WI, high signal intensity on T2WI, and no enhancement on contrast-enhanced imaging. The main pancreatic duct showed no dilation. The gallbladder wall showed no significant thickening, and no abnormal signals or enhancement were observed within the gallbladder on contrast-enhanced imaging. The spleen appeared normal in shape and signal intensity, with no abnormal enhancement on contrast-enhanced imaging. No significantly enlarged lymph nodes were observed in the abdominal aorta, mesenteric artery root, or hepatic hilum region. No ascites was present in the abdominal cavity.

Imaging findings suggest:

1. A space-occupying lesion in the right upper abdominal gallbladder fossa region with unclear boundaries from the left hepatic lobe, suspected to be hepatobiliary duct cell carcinoma (MT) of hepatic origin.
2. Compression and displacement of the adjacent gallbladder and gastric antrum, with abnormal perfusion in the adjacent hepatic parenchyma.
3. Abnormal enhancement foci in hepatic segments S2 and S8, suspected to be cavernous hemangiomas.
4. Multiple small cysts within the liver.
5. A small cyst in the pancreatic tail.

#### Ultrasound

**Figure 3:**
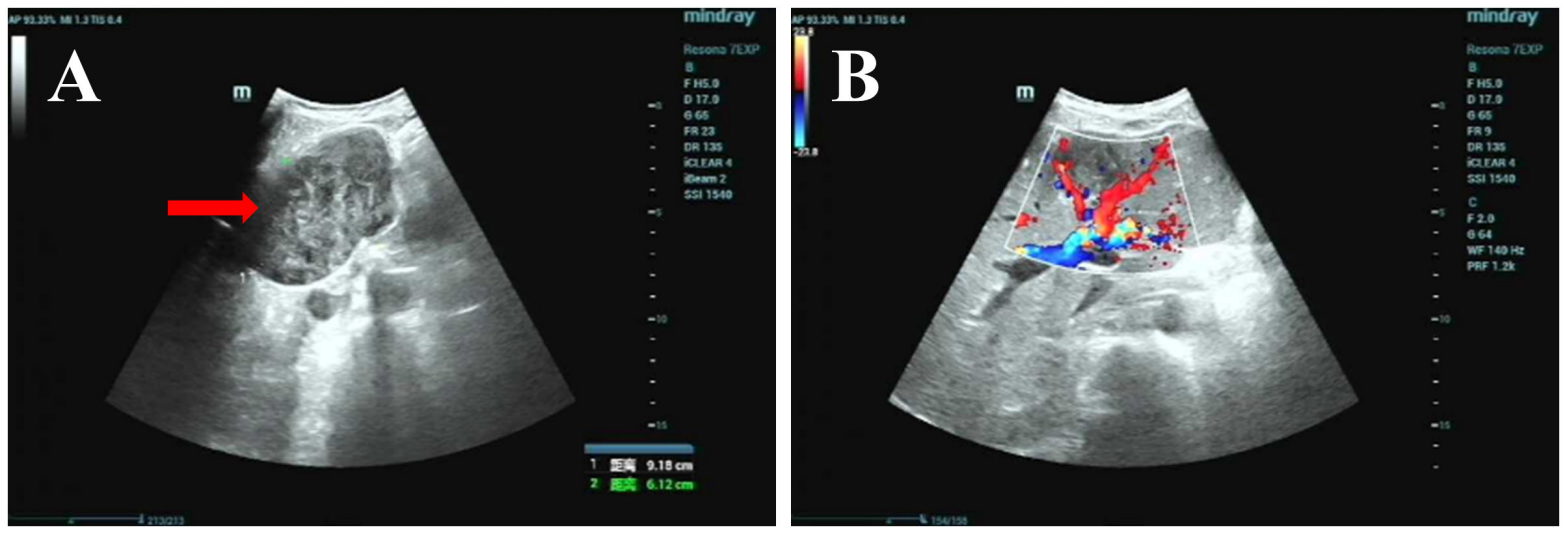
Ultrasound image: **(A)** Grayscale ultrasound of the left hepatic lobe, showing a 9.2×6.1 cm heterogeneous echoic mass with clear borders and irregular shape (red arrow); **(B)** Color Doppler ultrasound of the same region, showing vascular filling (red/blue signals) in surrounding tissues and no abnormal intratumoral blood flow (red arrow).

A liver-gallbladder-pancreas-spleen-kidney ultrasound was performed on December 16, 2024, and images are displayed in **Figure 3**. The liver appeared normal in shape and size, with uniform echogenicity of the parenchyma and a smooth capsule. A 9.2 x 6.1 cm heterogeneous echoic mass, characterized by alternating high and low echoes, was identified in the lower segment of the left lateral lobe and the left medial lobe. The mass had clear borders and an irregular shape. The gallbladder appeared compressed, and both the left hepatic vein and the left internal branch of the portal vein were compressed and displaced. Color Doppler imaging revealed filling in the vessels. No significant abnormalities were observed in the gallbladder, intra- and extrahepatic bile ducts, pancreas, spleen, or kidneys. The ultrasound findings suggest a solid space-occupying lesion within the liver, which is causing compression and displacement of the gallbladder, left hepatic vein, and left internal branch of the portal vein.

### Gastrointestinal tumor markers

Among the results of gastrointestinal tumor marker assays, alpha-fetoprotein (AFP) was measured at 8.09 μg/L, which is above the reference interval (0-6.66 μg/L). Carbohydrate antigen 72-4 (CA72-4) was 31.42 KIU/L, exceeding the reference interval (0–6 KIU/L). The remaining markers, including carcinoembryonic antigen (CEA, 3.09 μg/L), ferritin (250.00 μg/L), carbohydrate antigen 19-9 (CA19-9, <0.30 KU/L), and carbohydrate antigen 24-2 (CA24-2, <0.50 KIU/L), were within their respective reference ranges (**Table 2**).

**Table 2:** Gastrointestinal tumor markers results.

Serial number	Item name	Abbreviation	Abbreviation	Unit	Test method	Reference range
1	Alpha-fetoprotein	AFP	8.09↑	ug/L	Chemiluminescence	0-6.66
2	Carcinoembryonic antigen	CEA	3.09	ug/L	Chemiluminescence	0-5
3	Ferritin	FERRITIN	250.00	ug/L	Chemiluminescence	20-450
4	Carbohydrate Antigen CA19-9	CA19-9	<0.30	KU/L	Chemiluminescence	0-19
5	Carbohydrate Antigen CA72-4	CA72-4	31.42↑	KIU/L	Chemiluminescence	0-6
6	Carbohydrate Antigen CA24-2	CA24-2	<0.50	KIU/L	Chemiluminescence	0-10

### Diagnosis and differential diagnosis

Preoperative imaging studies, including CT and MRI, revealed progressive heterogeneous enhancement of the lesion, with delayed-phase capsular enhancement, consistent with the typical imaging features of cholangiocarcinoma. Anatomically, the tumor was located adjacent to the gastric antrum and gallbladder fossa, exerting compression on the gallbladder and obscuring its wall, which aligns with the common infiltration patterns of cholangiocarcinoma within the left hepatic lobe. The imaging report further described “the lesion with unclear boundaries from the left hepatic lobe and adjacent abnormal hepatic perfusion,” reinforcing the possibility of a tumor originating from the biliary system. Tumor marker testing showed significantly elevated CA72–4 levels, a marker often associated with bile duct or gastrointestinal tumors. Although CA72–4 lacks high specificity, its elevation, combined with the imaging findings and anatomical location, supports the diagnosis of cholangiocarcinoma. After differential diagnosis with other conditions, including liver adenoma, liver hemangioma, liver cancer, and liver abscess, the patient was considered to have a high likelihood of cholangiocarcinoma prior to surgery.

### Treatment

The patient underwent a left hepatic lateral segmentectomy on December 23, 2024. During the procedure, an exophytic tumor was identified, located in both the left lateral segment and a portion of the left medial segment of the liver, measuring approximately 10 cm in diameter. The entire left lateral segment, along with the tumor, was successfully resected. The intraoperative frozen section report indicated a diagnosis of “hepatic spindle cell tumor.” The excised specimen consisted of liver tissue measuring 13x7x4 cm. A tumor measuring 10x8x7 cm was noted on the surface of the liver. The tumor exhibited a gray-white solid consistency, was firm to the touch, and had well-defined borders from the surrounding liver tissue. The remaining liver tissue was sectioned into sheets, displaying a gray-red solid consistency, with no nodules present. The final pathological diagnosis confirmed the presence of a spindle cell tumor in the left hepatic lobe.

### Treatment outcomes, follow-up, and prognosis

#### Postoperative pathological examination

**Figure 4:**
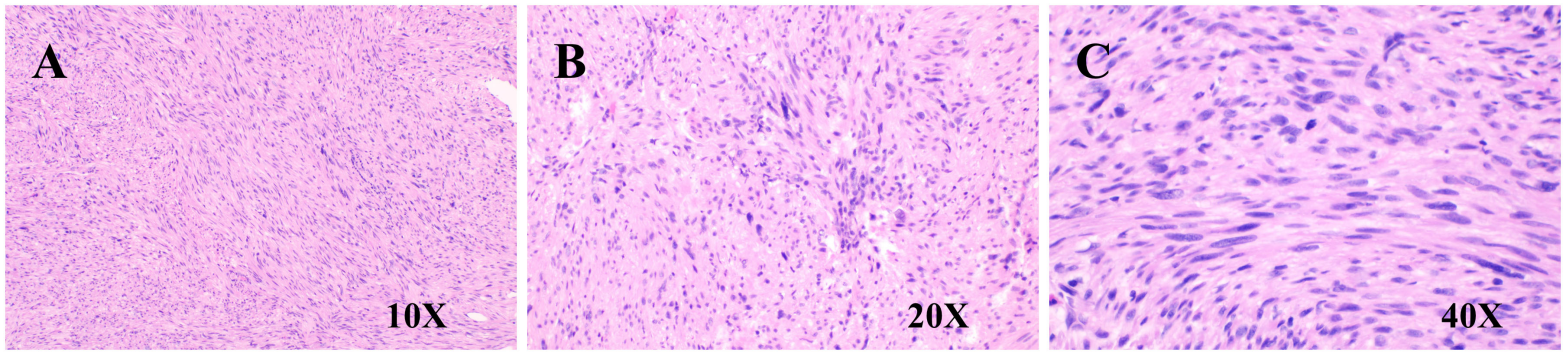
Pathological images. **(A)** H&E staining (10×, spindle cell morphology); **(B)** H&E staining (20×, cellular atypia); **(C)** H&E staining (40×, cellular atypia).

The immunohistochemical staining results for the left hepatic lobe and tumor are as follows (**Figure 4**): CD117 (sporadic +), DOGI (–), Vimentin (+), CD34 (vascular +), Actin (+), Desmin (+), S-100 (–), p63 (–), p53 (sporadic +), Ki67 (+, approximately 15%), and SDHB (+). Special staining: VG (+). Based on the morphological and immunohistochemical findings, the diagnosis is as follows: 1. Leiomyosarcoma, with tumor size measuring 10 cm × 8 cm × 7 cm, a smooth surface, and clear demarcation from the liver parenchyma. The tumor tissue does not invade the liver parenchyma. There is marked cellular atypia, coagulative necrosis, and a mitotic count > 10 per 10 high-power fields (HPF). 2. No tumor tissue was found at the liver parenchyma margin. 3. Focal fatty liver. 4. Chronic hepatitis, G3S1.

#### Follow-up and patient perspective

1–6 months postoperatively: Contrast-enhanced CT showed no recurrence or metastasis; liver function and tumor markers (AFP, CA72-4) returned to normal.

Patient perspective: “I was anxious when told it might be cancer, but the surgery went smoothly. Now I feel as healthy as before and can garden and walk daily. I’m willing to continue long-term follow-up to help other PHL patients.”

## Discussion

Primary hepatic leiomyosarcoma (PHL) is an exceedingly rare malignant tumor, accounting for approximately 0.2% of all liver malignancies (7–9). According to the existing literature, this tumor is extremely rare in clinical practice and is often misdiagnosed or overlooked, resulting in patients typically being diagnosed at an advanced stage. Studies have shown that the diagnosis of PHL is frequently delayed until the tumor has reached a larger size, which is associated with poor patient outcomes (8). Unlike other types of liver cancer, most literature reports suggest that the incidence of PHL is primarily concentrated in the middle-aged and elderly population. Some studies indicate that the incidence rate is slightly higher in females than in males, although male cases are also not uncommon (10). This gender disparity may be related to hormonal levels, genetic factors, or environmental influences; however, detailed mechanistic studies are currently lacking to further elucidate this phenomenon. Primary hepatic leiomyosarcoma primarily originates from smooth muscle cells in the liver and is typically associated with intrahepatic vessels, bile ducts, and the hepatic ligament (3). According to the literature, the pathological features of PHL generally include tumor cells with spindle or fusiform shapes, significant cellular atypia, and high proliferative activity. Common immunohistochemical markers include smooth muscle-specific antigens such as smooth muscle actin (SMA) and desmin (11). Furthermore, the histological features of the tumor may resemble those of other liver tumors, so a comprehensive pathological examination and immunohistochemical analysis are crucial for diagnosis to exclude other benign or malignant liver tumors (12).

PHL presents with a wide range of clinical manifestations. Common symptoms include right upper quadrant abdominal pain, weight loss, and decreased appetite. In some cases, a liver mass may be detected during a physical examination (13). Due to the nonspecific nature of its symptoms and imaging findings, PHL is often misdiagnosed as benign liver lesions, such as liver cysts, liver hemangiomas, or focal nodular hyperplasia (5). It can also be mistaken for other malignant liver tumors, including cholangiocarcinoma, hepatocellular carcinoma, or metastatic tumors (3). In certain instances, patients may exhibit abnormal liver function or other systemic symptoms, which can complicate the clinician’s ability to promptly identify the disease during the initial assessment (9). Therefore, clinicians should remain vigilant when encountering liver masses, particularly in high-risk populations, and consider the possibility of PHL.

The misdiagnosis of primary hepatic leiomyosarcoma (PHL) on imaging presents significant challenges for clinical decision-making. In this case, the patient was initially misdiagnosed with cholangiocarcinoma due to the tumor’s exophytic growth and its proximity to adjacent structures—an imaging scenario that is not uncommon. On imaging, leiomyosarcoma typically appears as an exophytic mass with progressive heterogeneous enhancement, whereas cholangiocarcinoma is characterized by rapid, transient enhancement and bile duct dilation. Imaging overlap between PHL and cholangiocarcinoma leads to frequent misdiagnosis. Based on this case and literature, key differentiating features are summarized in **Table 3**. PHL, on the other hand, often manifests on ultrasound as a well-defined or irregular hypoechoic mass, a feature that requires careful differentiation from other liver tumors in clinical practice (11).

**Table 3:** The key differentiating features between hepatic leiomyosarcoma and cholangiocarcinoma.

Feature	Cholangiocarcinoma	PHL
Bile Duct Dilation	Common (obstruction from duct wall invasion)	Rare (no duct involvement; our patient: CBD 0.4 cm, normal)
Enhancement Pattern	Peripheral arterial enhancement + gradual filling	Progressive heterogeneous enhancement + delayed capsular enhancement
Tumor Origin/Invasion	Bile duct epithelium (invades duct wall)	Hepatic smooth muscle (exophytic growth, compression only)
Necrosis	Peripheral (rare)	Central (common in large tumors ≥5 cm)

During CT scanning, PHL displays diverse imaging characteristics. Enhanced CT scans reveal heterogeneous enhancement of the tumor, with some cases showing central necrosis or scar-like areas, which typically present as low-density zones on imaging (14). One study reported that CT scans may reveal necrosis in the central portion of the tumor, with surrounding vessels being compressed, resulting in complex imaging features (3). Another case noted irregular enhancement patterns in the liver mass on contrast-enhanced CT scans, suggesting the possibility of malignant lesions (5).

MRI findings typically show low signal intensity on T1-weighted imaging (T1WI), high signal intensity on T2-weighted imaging (T2WI), and prominent marginal enhancement after contrast administration—findings confirmed by several studies (15). For instance, one study indicated that PHL appears on MRI as a well-defined, heterogeneous, low- or isodense mass with gradually increasing enhancement post-contrast (14). The nonspecific nature of these imaging findings often leads to confusion with other liver tumors, such as hepatocellular carcinoma, necessitating heightened clinical awareness (16).

Additionally, PHL can be mistaken for other types of liver tumors, such as hepatocellular carcinoma or metastatic liver cancer, particularly when the tumor is small or lacks typical characteristics (3). Consequently, the risk of imaging misdiagnosis remains high. To improve diagnostic accuracy, it is crucial to combine clinical manifestations and pathological examination results with imaging assessments (5). For cases with atypical imaging features, liver tissue biopsy, coupled with immunohistochemical analysis, serves as a key step in confirming the diagnosis of PHL (13).

SDHB (Succinate Dehydrogenase B) is a critical subunit of the mitochondrial respiratory chain complex II, playing an essential role in the tricarboxylic acid cycle and oxidative phosphorylation processes (17). The function of SDHB is closely linked to cellular energy metabolism; its deficiency or mutation can lead to the accumulation of succinate within cells, disrupting cellular energy balance and metabolic homeostasis. Research has shown that SDHB expression levels are strongly associated with tumor cell proliferation, migration, and invasion, particularly in the tumor microenvironment, where SDHB dysfunction may contribute to malignant transformation and tumor progression (18, 19).

Typically, positive SDHB expression correlates with benign tumor behavior. Studies have found that SDHB-positive tumors have a lower risk of malignant transformation, while SDHB-negative tumors tend to exhibit more invasive and metastatic characteristics. The loss of SDHB function has been linked to increased malignant potential and metastasis in tumors such as pheochromocytoma and paraganglioma (3, 20). However, the expression pattern and clinical significance of SDHB in soft tissue sarcomas, especially in primary hepatic leiomyosarcoma (PHL), remain inadequately explored.

Additionally, positive SDHB expression is inversely correlated with tumor biological characteristics, such as the Ki-67 cell proliferation index, suggesting that SDHB plays a crucial role in regulating tumor biological behavior. In this case, immunohistochemical analysis revealed positive SDHB expression, which sharply contrasts with gastrointestinal stromal tumors (GISTs), which typically exhibit SDHB deficiency. This finding indicates that PHL and GIST may differ in their SDHB deficiency pathways. The significance of SDHB positivity in PHL remains unclear and may be associated with distinct tumor-driving mechanisms, metabolic states, or potential prognostic differences (6).

While SDHB deficiency is often a marker of invasiveness in other tumors, the implication of SDHB positivity in PHL and whether it suggests more benign behavior remains to be confirmed through large-scale studies. The value of this case lies in suggesting that SDHB status could serve as a biologically significant marker in PHL. Future research should investigate its relationship with clinical and pathological characteristics in PHL patients, such as tumor grading, staging, recurrence, metastasis, and survival prognosis, and evaluate its potential for guiding targeted treatment strategies. These could include therapies targeting SDH-deficient or non-deficient pathways, providing a basis for personalized treatment approaches.

Surgical resection remains the primary and preferred treatment option for curing primary hepatic lymphoma (PHL) (7, 9). For patients with advanced disease who are not candidates for surgery, those with high-risk factors post-surgery (such as positive margins, high-grade tumors, large tumors, extensive necrosis), or those who experience recurrence or metastasis, systemic therapy becomes an essential treatment approach. Traditional chemotherapy regimens, often based on soft tissue sarcoma protocols (such as doxorubicin and irinotecan), are typically used for palliative care in advanced-stage patients. Some studies have also investigated their role in adjuvant therapy, but the overall efficacy is limited, and their impact on improving survival rates is not significant. According to a retrospective study, although chemotherapy may help control tumor growth, its effectiveness is often inferior to that of surgical resection, particularly when the tumor has advanced to a later stage (21). Currently, there is no established standard for adjuvant chemotherapy in PHL.

Emerging immune checkpoint inhibitors, such as PD-1/PD-L1 inhibitors, and targeted therapies have shown potential efficacy in various sarcoma subtypes, offering new treatment options for advanced soft tissue sarcoma patients (21). PD-1 inhibitors function by releasing the suppression of the immune system by tumor cells, thereby enhancing the body’s immune response and aiding in the elimination of tumor cells. In studies targeting other cancer types, PD-1 inhibitors like nivolumab and pembrolizumab have demonstrated significant improvements in patient survival rates and progression-free survival (22). However, efficacy data for these drugs in PHL, a rare subtype, remain extremely limited, primarily derived from case reports or small retrospective studies, and they are generally considered exploratory treatments after the failure of standard therapies. Although these therapies show promise in theory and in individual cases, whether they offer an advantage in improving overall survival in PHL remains to be validated through large-scale prospective studies.

This study has several limitations. First, it adopts a single-case design, which means its findings cannot be generalized to all PHL patients, and there is no statistical validation of SDHB’s prognostic role. Second, the follow-up period is short—six months is insufficient to evaluate the long-term recurrence risk of PHL, which typically requires 1–5 years of follow-up. Additionally, molecular testing was limited; no next-generation sequencing (NGS) was performed to explore SDHB-related genetic drivers of PHL. Furthermore, there are gaps in the existing literature, as few studies focus on human PHL and SDHB, which restricts the contextualization of the present study’s findings.

## Conclusions

This study presents a rare case of misdiagnosis of primary hepatic leiomyosarcoma (PHL) in a middle-aged male. By integrating imaging, pathological, and molecular characteristics, this case contributes to a deeper understanding of PHL and offers new insights into reducing the risk of misdiagnosis, ultimately enabling more personalized treatment approaches in clinical practice. Preoperative imaging revealed progressive heterogeneous enhancement and delayed capsular enhancement, characteristics that strongly overlap with those of cholangiocarcinoma, leading to an initial misdiagnosis. The correct diagnosis of PHL was confirmed through left lateral lobectomy, with the patient recovering well postoperatively. This case underscores the imaging similarities between PHL and cholangiocarcinoma, and for the first time, it highlights the significance of SDHB positive expression in PHL. This finding suggests that SDHB positivity may reflect relatively favorable tumor biology in PHL, potentially serving as a preliminary prognostic clue—however, its value in guiding targeted therapy requires validation in multi-center cohorts. Surgical resection remains the first-line curative option for localized PHL, with long-term follow-up essential for recurrence monitoring. Future research should focus on multi-center PHL cohorts to validate SDHB’s role and develop targeted therapies.

## Data Availability

The original contributions presented in the study are included in the article/supplementary material. Further inquiries can be directed to the corresponding author/s.
